# Molecular Mechanisms Underlying the Anticancer Properties of Pitavastatin against Cervical Cancer Cells

**DOI:** 10.3390/ijms25147915

**Published:** 2024-07-19

**Authors:** Ya-Hui Chen, Jyun-Xue Wu, Shun-Fa Yang, Yun-Chia Wu, Yi-Hsuan Hsiao

**Affiliations:** 1 Women’s Health Research Laboratory, Changhua Christian Hospital, Changhua 50006, Taiwan; 106317@cch.org.tw (Y.-H.C.); 183726@cch.org.tw (J.-X.W.); 2 Institute of Medicine, Chung Shan Medical University, Taichung 40201, Taiwan; ysf@csmu.edu.tw; 3 Department of Medical Research, Chung Shan Medical University Hospital, Taichung 40201, Taiwan; 4 Department of Obstetrics and Gynecology, Changhua Christian Hospital, Changhua 50006, Taiwan; 182890@cch.org.tw; 5 School of Medicine, Chung Shan Medical University, Taichung 40201, Taiwan; 6 College of Medicine, Kaohsiung Medical University, Kaohsiung 807378, Taiwan; 7 Department of Post-Baccalaureate Medicine, College of Medicine, National Chung Hsing University, Taichung 40227, Taiwan

**Keywords:** pitavastatin, cervical cancer, apoptosis, PI3K/AKT, MAPK

## Abstract

Cervical cancer ranks as the fourth most prevalent form of cancer and is a significant contributor to female mortality on a global scale. Pitavastatin is an anti-hyperlipidemic medication and has been demonstrated to exert anticancer and anti-inflammatory effects. Thus, the purpose of this study was to evaluate the anticancer effect of pitavastatin on cervical cancer and the underlying molecular mechanisms involved. The results showed that pitavastatin significantly inhibited cell viability by targeting cell-cycle arrest and apoptosis in Ca Ski, HeLa and C-33 A cells. Pitavastatin caused sub-G1- and G0/G1-phase arrest in Ca Ski and HeLa cells and sub-G1- and G2/M-phase arrest in C-33 A cells. Moreover, pitavastatin induced apoptosis via the activation of poly-ADP-ribose polymerase (PARP), Bax and cleaved caspase 3; inactivated the expression of Bcl-2; and increased mitochondrial membrane depolarization. Furthermore, pitavastatin induced apoptosis and slowed the migration of all three cervical cell lines, mediated by the PI3K/AKT and MAPK (JNK, p38 and ERK1/2) pathways. Pitavastatin markedly inhibited tumor growth in vivo in a cancer cell-originated xenograft mouse model. Overall, our results identified pitavastatin as an anticancer agent for cervical cancer, which might be expanded to clinical use in the future.

## 1. Introduction

Cervical carcinoma ranks as the fourth most common type of cancer and is a major contributor to cancer-related deaths among women globally [1]. The treatment strategies for cervical cancer include surgery, radiotherapy, chemotherapy and concurrent chemoradiotherapy [2]. Recent studies have shown poor outcomes in patients with metastatic [3] or recurrent cervical cancer [4]. Therefore, improving cervical cancer treatment and preventing recurrence or distant metastasis are essential.

Statins, or HMG-CoA reductase inhibitors, significantly reduce cholesterol levels and lower the risk of cardiovascular disease [5]. Various types of statin have been shown to decrease cancer risk among individuals diagnosed with type 2 diabetes mellitus [6]. Mechanisms of the anticancer activity of statins have been widely investigated, including oxidative stress, autophagy, cell-cycle arrest and apoptosis of cancer cells [5]. The use of statins in combination with traditional chemotherapy, particularly in ovarian cancer, has been examined to evaluate the effects. The effects of metformin and statins on ovarian cancer involve the tumor immune microenvironment, reversing T-cell exhaustion and improving immunotherapy outcomes in ovarian cancer patients [7]. There are large biological effects of statins on pancreatic cancer, non-cancer and stem cells. Their applicability in chemoadjuvant tumor therapy is supported in light of the selective toxicity of statins to cancer cells over stem cells and the effects on cancer cell spheroids. At sufficient concentrations for cancer cell growth inhibition, low efficacy of statins on non-tumor and stem cells was detected [8]. Pitavastatin has a favorable pharmacological profile, including a long half-life (up to 12 h) and minimal metabolism by cytochrome P450 (CYP) enzymes [9]. Pitavastatin calcium has been utilized in Japan since it was first approved there in 2003 and subsequently received approval for use in the United States six years later [10]. Several studies have shown the anticancer effects of pitavastatin. The effects of pitavastatin on human triple-negative breast cancer (TNBC) cell lines were investigated. Pitavastatin may trigger autophagy-dependent ferroptosis in TNBC cells through the mevalonate pathway [11]. Pitavastatin has been shown to induce apoptosis in oral cancer cells by activating the FOXO3a/PUMA apoptotic axis [12]. It suppressed liver cancer cells and inhibited tumor growth [13]. Pitavastatin demonstrated strong inhibitory effects on both lung cancer cells and angiogenesis [14]. The ability of pitavastatin to suppress stem cell growth in colon carcinoma has been documented [15]. Recent studies and current clinical trials have highlighted the repurposing of statins as anticancer drugs, and the mechanisms of the anti-neoplastic effects of statins include regulating the cell cycle through the p53-YAP axis, targeting apoptosis via the Bcl-2 signaling pathway, and modulating epigenetics by altering CpG methylation [16]. Regarding the potential anti-cancer properties of statins, further investigations are necessary to reveal their effects on cancer-promoting signaling pathways [16].

Apoptosis is a type of programmed cell death and is regarded as a carefully regulated process in which caspase activation plays a central role [17]. In cancer, the suppression of the apoptotic pathways occurs due to increased levels of antiapoptotic proteins or decreased expression of proapoptotic proteins. This can lead to resistance to commonly employed anticancer treatments, as indicated by specific alterations [18]. The activation of the apoptotic pathway holds great potential for anticancer therapy, as it exhibits promising anticancer activity [18]. Apoptosis evasion is a hallmark of all types of cancer. Targeting and activating the apoptotic pathway is a promising nonsurgical treatment for various cancers [17].

The PI3K/Akt signaling cascade is integral to various biological processes and is aberrantly activated in many malignancies. This dysregulation is implicated in both the emergence and advancement of neoplasms [19]. Discovering effective inhibitors of this mechanism may improve the survival rate of cancer patients and is therefore an important approach. There are various challenges and restrictions when cancer treatment involves approaches that target the PI3K/Akt signaling pathway. These approaches include inhibitors of PI3K, inhibitors that target both PI3K and mTOR, inhibitors of Akt and inhibitors of the catalytic site of the mTOR complex. Among them, one of the most effective therapeutic strategies is to suppress the PI3K/Akt pathway, which is activated in cancer [20].

Mitogen-activated protein kinase (MAPK) signaling is a remarkable mechanism that coordinates and controls various cellular processes, including cell proliferation and differentiation, apoptosis, and cell responses when cells are under stress [21]. MAPKs play a significant role in tumor progression and are frequently implicated in drug resistance. Within the MAPK family, numerous kinases coordinate multiple important signaling pathways that undergo alterations and pathological deregulation in cancer [22]. The signal-regulated kinases ERK1 and ERK2 play significant roles in cellular signaling, and proper expression of ERK is essential for the development and progression of cancer [21]. Additionally, protein kinase B (Akt) is central to several cellular mechanisms, including cell metabolism, cell growth, suppression of apoptosis, and angiogenesis. In the field of oncology therapy, the Akt pathway significantly affects cell growth and promotes cell survival [23]. On the other hand, MAPK pathways are essential for regulating molecular mechanisms, including cell proliferation, cell survival, and cell differentiation, while dysregulation of these pathways may lead to metastasis [24]. The potential for targeting the MAPK signaling pathway in the development of novel cancer therapies is being investigated in many cancer types. However, the underlying mechanisms are still unclear, especially for cervical cancer. Therefore, this study sought to investigate the potential antitumor effects and molecular mechanisms of pitavastatin on cervical cancer cells.

## 2. Results

### 2.1. Pitavastatin Has a Dose-Dependent Effect on Suppressing Cell Growth and Colony Formation

**Figure 1:**
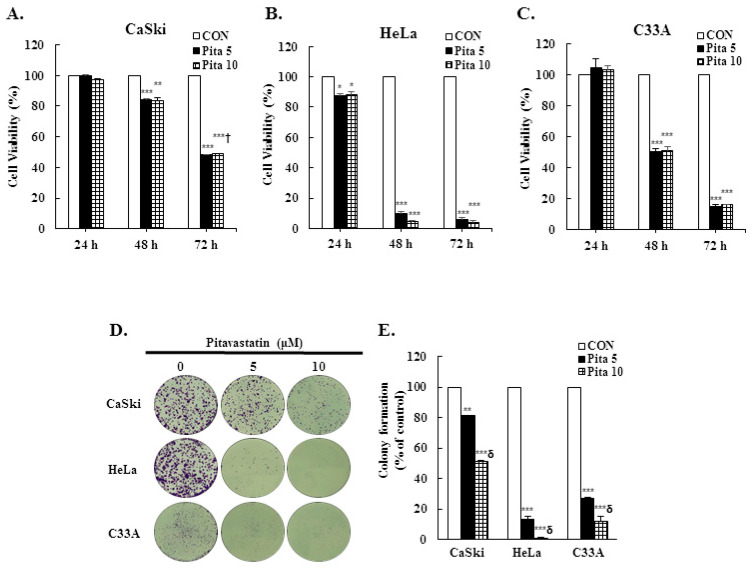
Pitavastatin inhibited cervical cancer cell viability and colony formation in a dose-dependent manner. (**A**–**C**) After treatment with pitavastatin (0, 5 and 10 μM) for 24, 48 and 72 h, the viability of Ca Ski, HeLa and C-33 A cells was measured using a CCK-8 assay. (**D**,**E**) Colony formation of Ca Ski, HeLa and C-33 A cells treated with pitavastatin (0, 5 and 10 μM) for 48 h. The colony formation ability of Ca Ski, HeLa and C-33 A cells was quantified by measuring the absorbance of the solution obtained by crystal violet, at 100× magnification, scale bar = 200 μm. The values represent the means ± SDs of three replicates. * *p* ˂ 0.05, ** *p* ˂ 0.01, *** *p* ˂ 0.001 compared with the CON group; ^†^ *p* ˂ 0.05, ^δ^ *p* ˂ 0.001 compared with the Pita 5-treated group. CON—0.1% DMSO; Pita 5—5 μM pitavastatin; Pita 10—10 μM pitavastatin.

To assess the anticancer effect of pitavastatin on cervical cancer, Ca Ski, HeLa and C-33 A cells were treated with various concentrations of pitavastatin (0–10 μM) for 24, 48 and 72 h, and cell viability and colony formation were assayed using the CCK-8 and crystal violet staining methods, respectively. Figure 1A–C shows that pitavastatin significantly inhibited the viability of Ca Ski, HeLa and C-33 A cells in a dose- and time-dependent manner (*p* < 0.05), although 5 μM or 10 μM pitavastatin did not affect the viability of Ca Ski or C-33 A cells after 24 h. Compared with the control treatment, pitavastatin decreased the colony formation ability of cervical cancer cells in a dose-dependent manner. Treatment with 5 μM or 10 μM pitavastatin decreased colony numbers of Ca Ski cells by 19% and 49%, respectively, decreased colony numbers of HeLa cells by 87% and 99%, respectively, and decreased colony numbers of C-33 A cells by 73% and 88%, respectively (Figure 1D,E, *p* < 0.05). Thus, these results indicated that pitavastatin has antiproliferative potential in cervical cancer cells. 

### 2.2. Pitavastatin Promotes Cell Apoptosis and Cell Cycle Arrest

**Figure 2:**
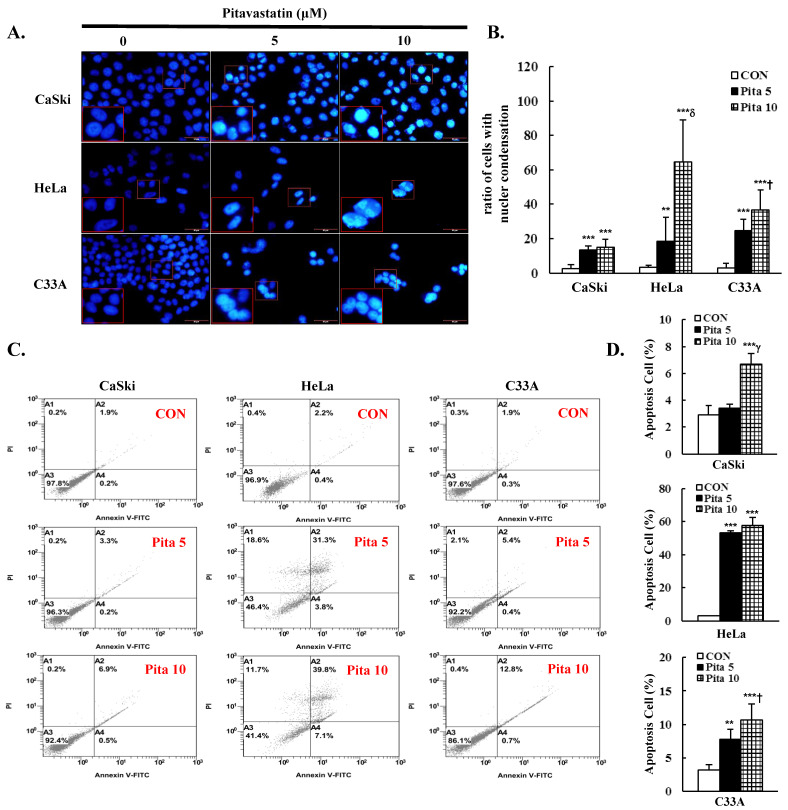
Pitavastatin induces cell apoptosis in cervical cancer cell lines after treatment with pitavastatin (0, 5 and 10 μM) for 48 h, (**A**) the nuclear condensation of Ca Ski, HeLa and C-33 A cells was measured with DAPI staining and analyzed by fluorescence microscopy at 400× magnification; scale bar = 50 μm. (**B**) The relative density of the ratio of cells with nuclear condensation. (**C**) Cells were stained with Annexin V/PI and analyzed by flow cytometry. (**D**) Quantitative relative density of the percentage of apoptotic cells (including cells in the early and late stages). The values represent the means ± SDs of three replicates. ** *p* ˂ 0.01, *** *p* ˂ 0.001 compared with the CON group; ^†^ *p* ˂ 0.05, ^γ^ *p* ˂ 0.01, ^δ^ *p* ˂ 0.001 compared with the Pita 5-treated group. CON—0.1% DMSO; Pita 5—5 μM pitavastatin; Pita 10—10 μM pitavastatin.

**Figure 3:**
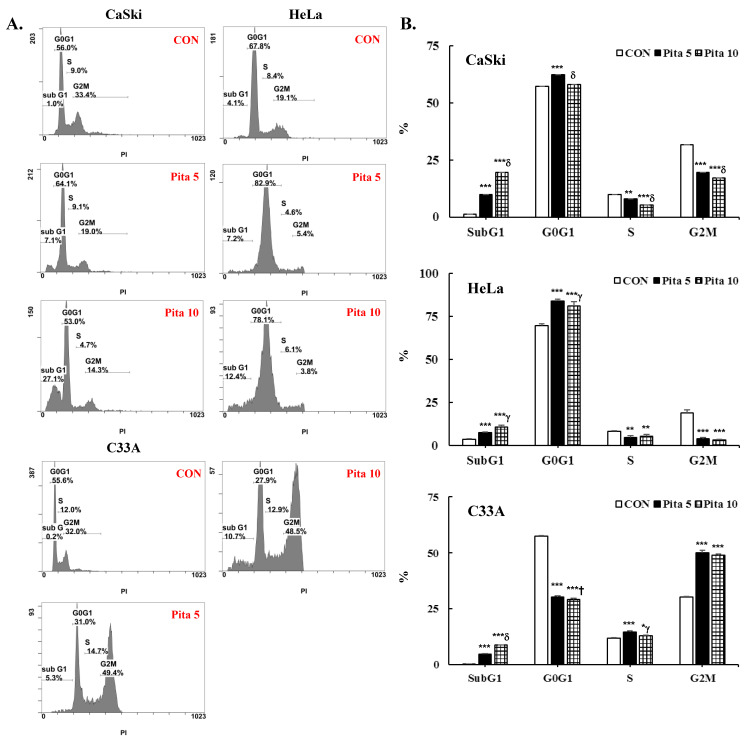
Pitavastatin leads to sub-G1, G0/G1 and G2/M cell cycle arrest in cervical cancer cell lines. (**A**) The cell cycle distribution of Ca Ski, HeLa and C-33 A cells treated with pitavastatin (0, 5 and 10 μM) for 48 h was analyzed by flow cytometry. (**B**) Quantitation of the cell cycle distribution (sub-G1, G0/G1, S and G2/M). The values represent the means ± SDs of three replicates. * *p* ˂ 0.05, ** *p* ˂ 0.01, *** *p* ˂ 0.001 compared with the CON group; ^†^ *p* ˂ 0.05, ^γ^ *p* ˂ 0.01, ^δ^ *p* ˂ 0.001 compared with the Pita 5-treated group. CON—0.1% DMSO; Pita 5—5 μM pitavastatin; Pita 10—10 μM pitavastatin.

The inhibition of cell proliferation by pitavastatin is connected with cell apoptosis and cell cycle arrest. Cell apoptosis and the cell cycle were measured through cytometry using Annexin V/PI and PI/RNase staining methods, respectively. In addition, the nuclear dye DAPI was used to stain the pitavastatin-treated cervical cancer cells for fluorescence microscopy. As shown in Figure 2A,B, pitavastatin treatment significantly increased nuclear condensation and nuclear fragmentation in a dose-dependent manner (Ca Ski cells, 2.5 ± 2.2 vs. 13.6 ± 2.0 vs. 14.9 ± 4.5; HeLa cells, 3.3 ± 1.20 vs. 18.5 ± 14.0 vs. 64.6 ± 24.3; C-33 A cells, 2.8 ± 2.8 vs. 24.5 ± 6.8 vs. 36.7 ± 11.7, *p* < 0.05) and significantly decreased the cell number. Compared with the control group, the highest concentrations of pitavastatin significantly increased the percentage of apoptotic cells (Ca Ski cells, 6.7 ± 0.8% vs. 2.9 ± 0.7%; HeLa cells, 57.6 ± 5.0% vs. 2.9 ± 0.3%; C-33 A cells, 10.7 ± 2.4% vs. 3.2 ± 0.8%; Figure 2C,D, *p* < 0.05). According to the cell cycle distribution assay, the highest concentration of pitavastatin increased the proportion of Ca Ski cells in the sub-G1 phase from 1.2 ± 0.1% to 19.5 ± 0.8%. Moreover, the percentage of sub-G1-phase cells increased from 3.6 ± 0.3% to 10.6 ± 1.1%, and the percentage of G0/G1-phase cells increased from 69.4 ± 1.3% to 81.2 ± 2.2% in HeLa cells. The percentages of sub-G1- and G2/M-phase cells were also significantly increased from 0.3 ± 0.1% to 8.8 ± 0.1% and from 30.4 ± 0.1% to 49.1 ± 0.5%, respectively, in C-33 A cells (Figure 3, *p* < 0.05). Our results revealed that pitavastatin-mediated cervical cancer cell proliferation inhibition occurred via the induction of apoptosis and cell cycle arrest.

### 2.3. Pitavastatin Inhibits Cervical Cancer Cell Migration

**Figure 4:**
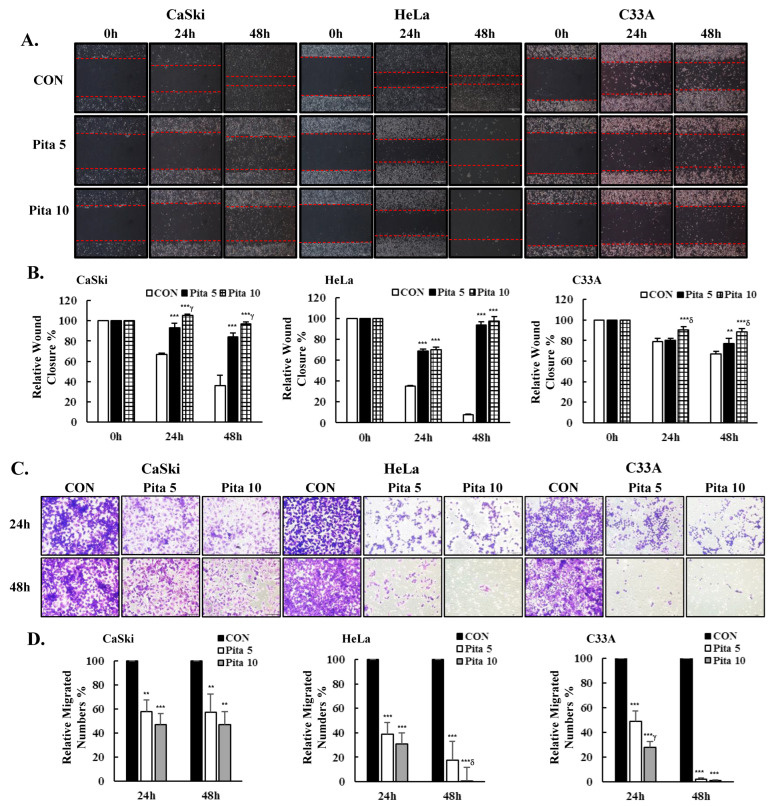
Pitavastatin inhibits the migration of cervical cancer cell lines. (**A**) Ca Ski, HeLa and C-33 A cells were treated with pitavastatin (0, 5 and 10 μM) and photographed at 0, 24 and 48 h to measure the extent of wound closure, usually using a 200-μL sterile pipette tip, leaving two wound edges (red lines) separated by a void. (**B**) Semi-quantitative analysis of the relative wound closure of Ca Ski, HeLa and C-33 A cells were performed by measuring the width of the wounds. (**C**) Ca Ski, HeLa and C-33 A cells migration capacity were evaluated by using 24-well Boyden chamber. (**D**) Quantitative relative density of the percentage of migration numbers. The values represent the means ± SDs of three replicates. ** *p* ˂ 0.01, *** *p* ˂ 0.001 compared with the CON group; ^γ^ *p* ˂ 0.01, ^δ^ *p* ˂ 0.001 compared with the Pita 5-treated group. CON—0.1% DMSO; Pita 5—5 μM pitavastatin; Pita 10—10 μM pitavastatin.

Next, to investigate whether pitavastatin could inhibit the migration of cervical cancer cells, we performed a wound healing and transwell migration assay. We observed that cells in the pitavastatin treatment group had a poorer cell migration capability, as their relative wound healing speed was lower than that in the untreated group at both 24 and 48 h. Pitavastain (5 μM or 10 μM) effectively reduced the wound closure speed by 48% and 61%, respectively, in Ca Ski cells and by 86% and 90%, respectively, in HeLa cells, as well as by 10% and 22%, respectively, in C-33 A cells compared with that in the control group at 48 h (Figure 4A,B, *p* < 0.05). Similarly, treatment with 5 μM or 10 μM pitavastain also significantly decreased the number of migration cells by 43% and 53%, respectively, in Ca Ski cells and by 82% and 99%, respectively, in HeLa cells, as well as by 98% and 99%, respectively, in C-33 A cells compared with that in the control group (Figure 4C,D, *p* < 0.05). Therefore, pitavastatin can inhibit the migration of cervical cancer cells and may further prevent their metastasis.

### 2.4. Pitavastatin Reduces the Mitochondrial Membrane Potential (∆ψm) and Activates Mitochondria-Mediated Apoptosis

**Figure 5:**
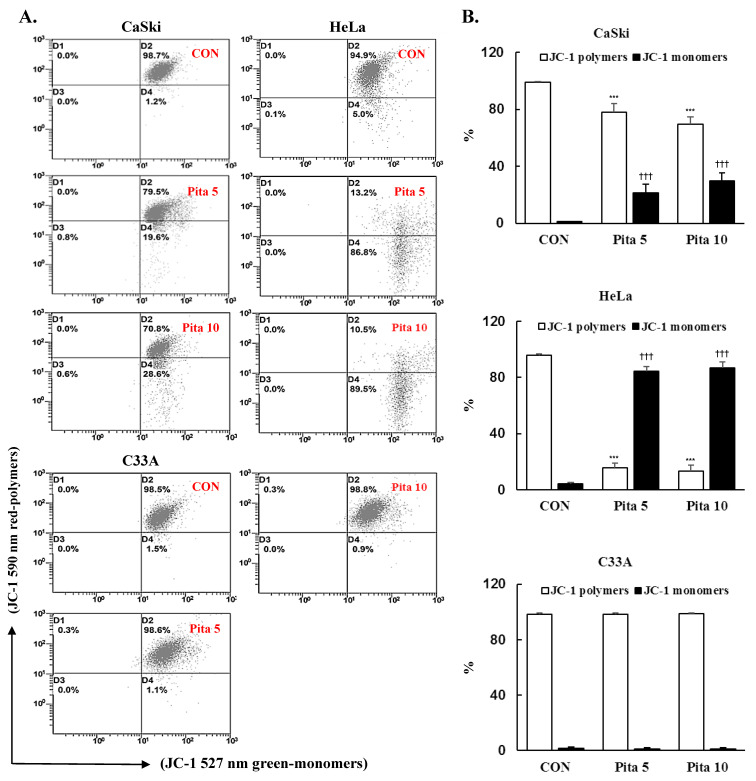
Pitavastatin reduces the mitochondrial membrane potential (∆ψm**)** in cervical cancer cell lines. Ca Ski, HeLa and C-33 A cells were treated with pitavastatin (0, 5 and 10 μM) for 48 h and stained with JC-1 dye. (**A**) Flow cytometry analysis showing the distribution of JC-1 green-positive cells with lower ∆ψm. (**B**) Quantitative analysis of the percentage of red-polymer and green-monomer fluorescence. The values represent the means ± SDs of three replicates. *** *p* ˂ 0.001 and ^†††^ *p* ˂ 0.001 compared with the CON with polymers- or CON with monomers-treated group. CON—0.1% DMSO; Pita 5—5 μM pitavastatin; Pita 10—10 μM pitavastatin.

**Figure 6:**
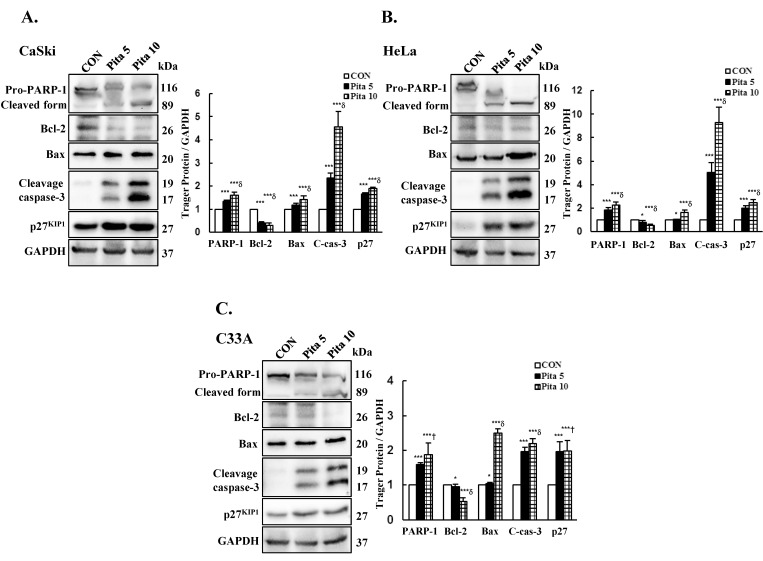
Pitavastatin causes cell apoptosis by activating apoptosis-related proteins in cervical cancer cell lines. Ca Ski (**A**), HeLa (**B**) and C-33 A (**C**) cells were treated with pitavastatin (0, 5 and 10 μM) for 48 h, and the PARP-1, Bcl-2, Bax, cleaved caspase 3 and p27^KIP1^ levels were measured via Western blotting, with GAPDH serving as an internal control. The quantitative results are shown in the bottom plot. The values represent the means ± SDs of three replicates. * *p* ˂ 0.05, *** *p* ˂ 0.001 compared with the CON group; ^†^ *p* ˂ 0.05, ^δ^ *p* ˂ 0.001 compared with the Pita 5-treated group. CON—0.1% DMSO; Pita 5—5 μM pitavastatin; Pita 10—10 μM pitavastatin.

The mitochondrial membrane potential (MMP) is an essential parameter for regulating mitochondrial function, and the collapse of ∆ψm often occurs during the process of apoptosis. Thus, ∆ψm was measured using JC-1 dye after pitavastatin treatment. As shown in Figure 5, Ca Ski and HeLa cells treated with 5 μM or 10 μM pitavastatin showed significant depolarization of the MMP, as indicated by the increase in the green fluorescence intensity, which was increased by 20% and 29% in Ca Ski cells and by 80% and 82% in HeLa cells, respectively. While no change in the green fluorescence intensity was observed in C-33 A cells compared with that in the control cells. These results indicated that pitavastatin-induced apoptosis was associated with the depolarization of the MMP in Ca Ski and HeLa cells but not in C-33 A cells. To clarify the role of pitavastatin in inducing apoptosis, the expression levels of PARP-1, Bcl-2, Bax and cleaved caspase 3 were also investigated by Western blotting. The results indicated that the highest concentration of pitavastatin significantly increased the protein expression levels of PARP-1, Bax and cleaved caspase 3 (Ca Ski cells, ratio of control: 1.60 ± 0.15 vs. 1.43 ± 0.14 vs. 4.57 ± 0.65; HeLa cells, ratio of control: 2.26 ± 0.27 vs. 1.62 ± 0.21 vs. 9.26 ± 1.35; C-33 A cells, ratio of control: 1.88 ± 0.34 vs. 2.50 ± 0.12 vs. 2.20 ± 0.13, respectively), while the protein expression levels of Bcl-2 (Ca Ski cells, ratio of control: 0.29 ± 0.11; HeLa cells, ratio of control: 0.52 ± 0.10; C-33 A cells, ratio of control: 0.52 ± 0.12, respectively; Figure 6, *p* < 0.05) decreased in cervical cancer cells compared with those in the control groups. Additionally, the protein expression of the cyclin-dependent kinase inhibitor p27^KIP1^, which can inhibit cell cycle progression and promote cell apoptosis, was significantly increased in Ca Ski, HeLa and C-33 A cells (ratios of control: 1.91 ± 0.05 vs. 2.45 ± 0.28 vs. 1.99 ± 0.29, respectively) treated with 10 μM pitavastatin. These results clearly suggested that pitavastatin-induced apoptosis occurred through the mitochondria-mediated intrinsic signaling pathway and was correlated with p27^KIP1^ expression. 

### 2.5. Pitavastatin-Induced Apoptosis Specifically Targets the AKT and MAPK Pathways

**Figure 7:**
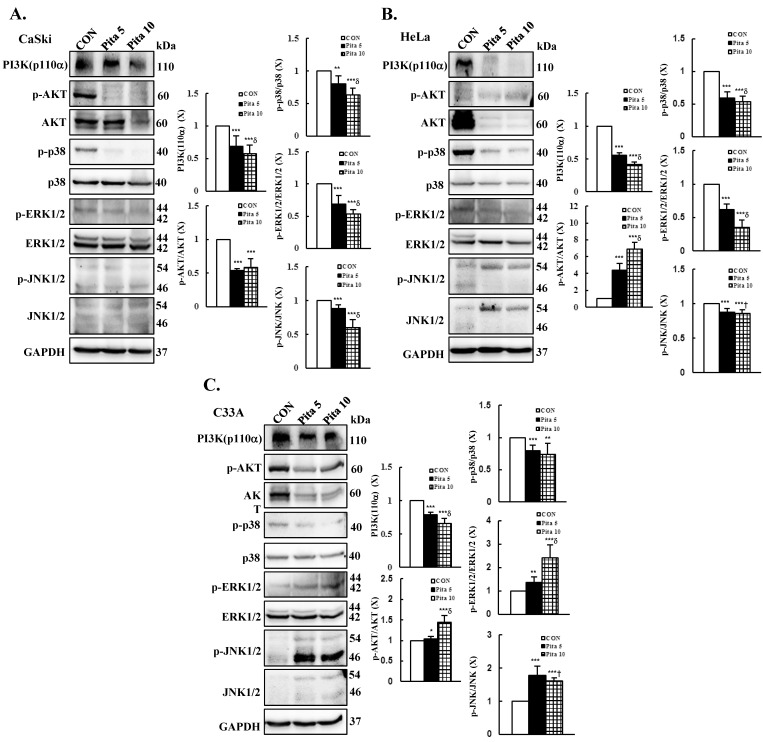
The PI3K/AKT and MAPK pathways are involved in pitavastatin-induced apoptosis regulation in cervical cancer cell lines. After treatment with pitavastatin (0, 5 and 10 μM) for 48 h, total and phosphorylated PI3K (110α), AKT, p38, ERK1/2 and JNK1/2 levels in Ca Ski (**A**), HeLa (**B**) and C-33 A (**C**) cells were measured via Western blotting, with GAPDH serving as an internal control. The quantitative results are shown in the bottom plot. The values represent the means ± SDs of three replicates. * *p* ˂ 0.05, ** *p* ˂ 0.01, *** *p* ˂ 0.001 compared with the CON group; ^†^ *p* ˂ 0.05, ^δ^ *p* ˂ 0.001 compared with the Pita 5-treated group. CON—0.1% DMSO; Pita 5—5 μM pitavastatin; Pita 10—10 μM pitavastatin.

The PI3K/AKT and MAPK pathways are the key signaling cascades that maintain cancer cell proliferation, evading apoptosis and sustaining angiogenesis [25,26]. Moreover, our recent study showed that PI3K/AKT and MAPK pathway-associated protein expressions are involved in modulating cervical cancer cell apoptosis and migration [27,28,29]. To investigate the effects of pitavastatin on the PI3K/AKT and MAPK pathways, we examined the phosphorylation of PI3K, AKT, JNK, p38, and ERK by Western blotting. As shown in Figure 7A, significant decreases in the levels of phosphorylated PI3K (p110α), AKT, p38, ERK1/2 and JNK1/2 proteins were detected in Ca Ski cells treated with a higher concentration of pitavastatin compared with the control group (ratios of control: 0.57 ± 0.14 vs. 0.58 ± 0.13 vs. 0.63 ± 0.11 vs. 0.53 ± 0.07 vs. 0.60± 0.12, respectively, *p* < 0.05). Similar results were observed for HeLa cells; pitavastatin treatment significantly decreased PI3K (p110α) and p38, ERK1/2 and JNK1/2 phosphorylation (ratios of control: 0.42 ± 0.03 vs. 0.54 ± 0.08 vs. 0.35 ± 0.11 vs. 0.86 ± 0.05, respectively; Figure 7B, *p* < 0.05), but *p*-AKT expression was significantly increased (ratio of control: 6.92 ± 0.77) compared with that in the control group. In C-33 A cells, PI3K (p110α) and p-p38 were significantly decreased after treatment with pitavastatin, whereas the levels of the phosphorylated proteins AKT, ERK1/2 and JNK1/2 were significantly increased (5 and 10 μM pitavastatin, ratios of control: 0.66 ± 0.07 vs. 0.74 ± 0.17 vs. 1.45 ± 0.16 vs. 2.40 ± 0.57 vs. 1.62 ± 0.08, respectively; Figure 7C, *p* < 0.05). Taken together, these findings suggested that pitavastatin induces cervical cancer cell apoptosis by specifically targeting the AKT and MAPK pathways.

### 2.6. Caspase 3-Dependent Apoptosis Pathway Is Activated by Pitavastatin

**Figure 8:**
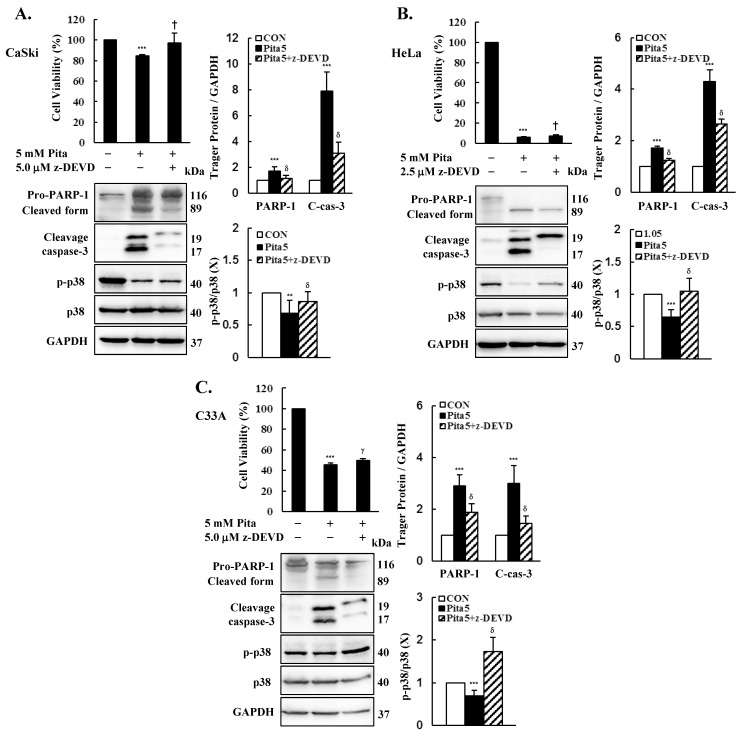
The caspase 3-dependent apoptosis pathway is activated by pitavastatin in cervical cancer cell lines. Ca Ski, HeLa and C-33 A cells were pretreated with z-DEVD-fmk (2.5 or 5.0 μM) for 2 h and then treated with pitavastatin (5 μM) for 48 h. Ca Ski (**A**), HeLa (**B**) and C-33A (**C**) cell viability were measured through the CCK-8 assay. PARP-1, cleaved caspase 3, p-p38 and p38 levels were detected by Western blotting. GAPDH was used as an internal control. The quantitative results are shown in the bottom plot. The values represent the means ± SDs of three replicates. ** *p* ˂ 0.01, *** *p* ˂ 0.001 compared with the CON group; ^†^ *p* ˂ 0.05, ^γ^ *p* ˂ 0.01, ^δ^ *p* ˂ 0.001 compared with the Pita 5-treated group. CON—0.1% DMSO; Pita 5—5 μM pitavastatin; Pita 5+ z-DEVD—5 μM pitavastatin + 2.5 μM z-DEVD or 5 μM pitavastatin + 5.0 μM z-DEVD.

To further validate the correlation between the caspase-dependent apoptosis pathway and pitavastatin-induced apoptosis, we used the caspase 3 inhibitor z-DEVD-fmk in further experiments. We observed that pitavastatin and z-DEVD-fmk co-treatment reversed the decrease in cell viability in Ca Ski, HeLa and C-33 A cells compared with that in the pitavastatin-only group (Ca Ski cells, 84.3 ± 1.5% vs. 97.0 ± 9.5%; HeLa cells, 6.3 ± 0.8% vs. 7.4 ± 1.2%; C-33 A cells, 45.4 ± 2.0% vs. 49.9 ± 1.5%; Figure 8, *p* < 0.05). Moreover, Western blot results revealed that pitavastatin and z-DEVD-fmk co-treatment also inhibited the PARP-1 and cleavage caspase 3 protein expression levels, which were upregulated in the pitavastatin-only group (Ca Ski cells, ratio of control: 1.73 ± 0.36 vs. 1.16 ± 0.25, 7.93 ± 1.46 vs. 3.10 ± 0.88; HeLa cells, ratio of control: 1.71 ± 0.09 vs. 2.14 ± 0.07, 4.30 ± 0.45 vs. 2.66 ± 0.17; C-33 A cells, ratio of control: 2.91 ± 0.42 vs. 1.89 ± 0.33, 3.00 ± 0.69 vs. 0.46 ± 0.28, respectively), and p-p38 protein showed an enhanced expression level in comparison with the pitavastatin-only group (Ca Ski cells, ratio of control: 0.68 ± 0.20 vs. 0.86 ± 0.16; HeLa cells, ratio of control: 0.67 ± 0.16 vs. 1.03 ± 0.25; C-33 A cells, ratio of control: 0.75 ± 0.16 vs. 1.86 ± 0.36, respectively, *p* < 0.05). These results clearly indicated that pitavastatin suppresses cervical cancer cell proliferation by activating the caspase 3-dependent apoptosis pathway.

### 2.7. Pitavastatin Inhibits C-33 A Xenograft Tumor Growth

**Figure 9:**
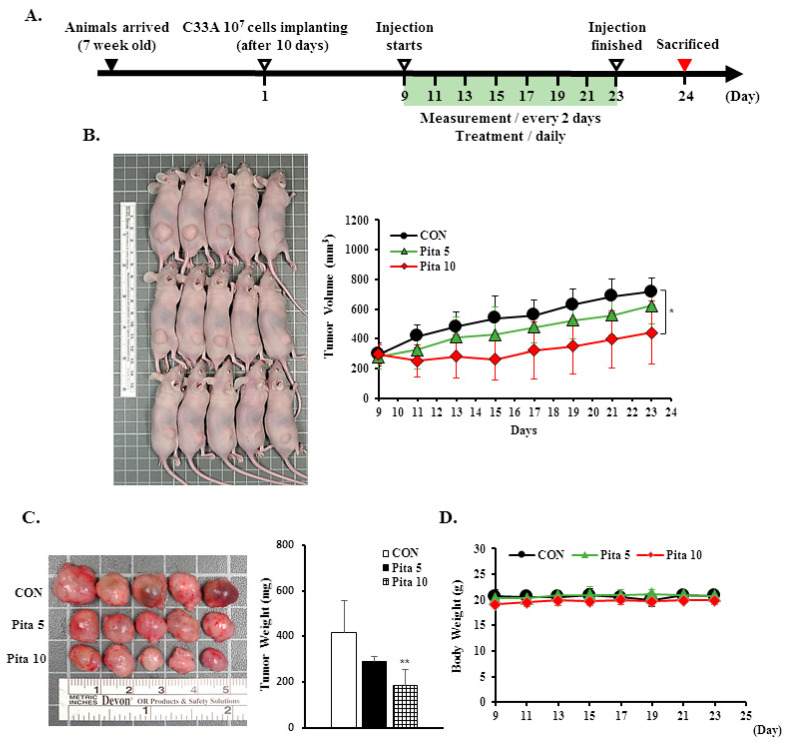
Pitavastatin effectively suppresses C-33 A xenograft tumor growth. C-33 A cells (1 × 107 cells) were injected subcutaneously into the right flank of BALB/c nude mice, and pitavastatin at 5 mg/kg or 10 mg/kg was then given by intraperitoneal injection every day to the mice. (**A**) Schematic representation of the experiment. (**B**) Representative images of tumors in C-33 A xenograft nude mice and tumor volume, as well as tumor weight (**C**) and body weight (**D**). The quantitative results are shown in the bottom plot. The values represent the means ± SDs (n = 8/group). * *p* < 0.05, ** *p* < 0.01 compared with the CON group. CON—control; Pita 5—5 mg/kg pitavastatin; Pita 10—10 mg/kg pitavastatin.

**Figure 10:**
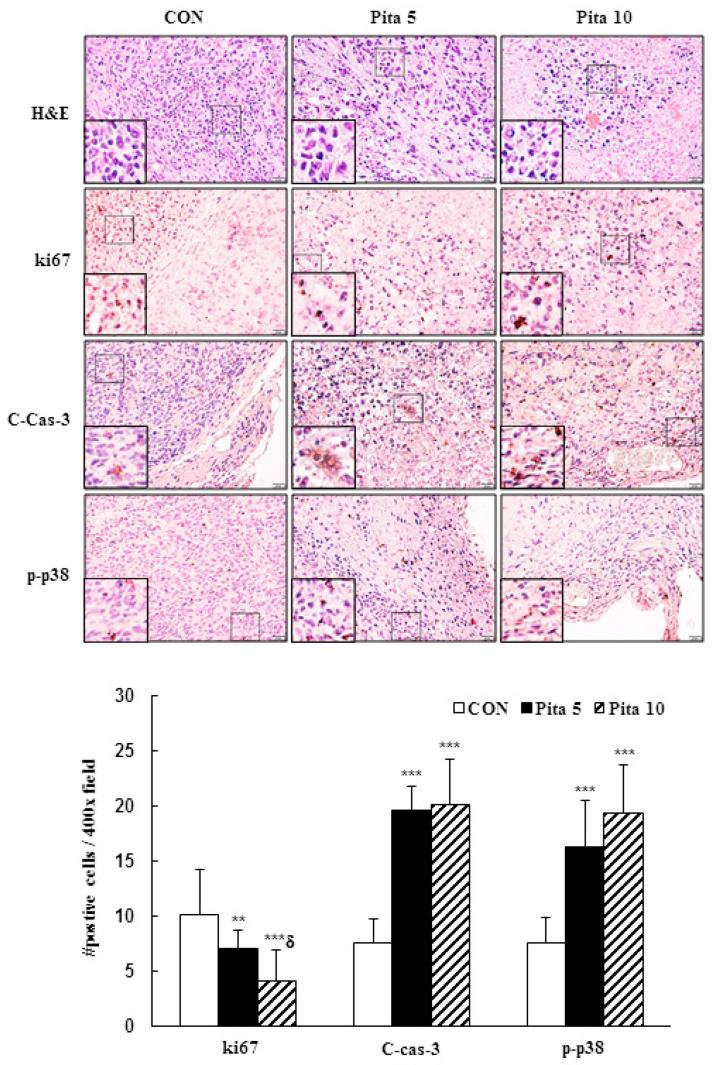
Pitavastatin effectively reduces ki67 expression and enhances cleaved caspase 3 and p-p38 expression in mammary tumor tissues from C-33 A xenograft nude mice. Mammary tissues were collected from pitavastatin-treated and untreated mice (*n* = 8/group). Representative histological sections of tumor tissue were stained with hematoxylin and eosin, a proliferation marker (ki67), an apoptosis marker (cleaved caspase 3), and p-p38 (shown as brown staining; H&E, 200× magnification, scale bar = 50 μm; immunohistochemical analysis, 400× magnification, scale bar = 20 μm). The quantitative results are shown in the bottom plot. The values represent the means ± SDs. ** *p* ˂ 0.01, *** *p* ˂ 0.001 compared with the CON group; ^δ^ *p* ˂ 0.001 compared with the Pita 5-treated group. CON—control; Pita 5—5 mg/kg pitavastatin; Pita 10—10 mg/kg pitavastatin.

To confirm the in vitro results, a C-33 A xenograft tumor model was established in BALB/c nude mice to determine the antiproliferative effect of pitavastatin in vivo. The timeline diagram of this experiment is presented in Figure 9A. Treatment of the C-33 A xenograft tumor model with the highest concentration of pitavastatin (10 mg/kg) significantly inhibited tumor growth compared with that in the control group, although no significant difference was observed in the lower dose of pitavastatin (5 mg/kg) group. After treatment for 14 days, the tumor volumes of the C-33 A xenograft tumors were 717.5 ± 89.3 mm^3^ vs. 624.0 ± 124.5 mm^3^ vs. 440.9 ± 210.7 mm^3^ in the control group and in the pitavastatin (5 mg/kg)- and pitavastatin (10 mg/kg)-treated groups, respectively, and the tumor growth inhibition rates were 13.0% and 38.6%, respectively (Figure 9B, *p* < 0.05). The average animal tumor weight was significantly lower in the pitavastatin (5 mg/kg)- and pitavastatin (10 mg/kg)-treated groups (289.8 ± 23.9 mg vs. 184.4 ± 69.6 mg, respectively) than in the control group (415.8 ± 140.3 mg, Figure 9C, *p* < 0.05). During the experimental period, the animal body weights did not significantly differ (20.8 ± 1.0 g vs. 20.8 ± 0.7 g vs. 19.9 ± 0.8 g, respectively), and the survival rates of the mice in the control and pitavastatin-treated groups were also 100%, suggesting that the dosage of pitavastatin did not cause drug toxicity to the host (Figure 9D). Moreover, we found that the rates of sparse tumor cellularity and apoptosis in the pitavastatin-treated groups were significantly greater than those in the control. Similarly, consistent with the results of this in vitro study, pitavastatin at 5 mg/kg and 10 mg/kg effectively reduced ki67 protein expression and enhanced cleaved caspase 3 and p38 phosphorylation protein expression in C-33 A xenograft tumor mice compared with the control group (Ki67, 10.1 ± 4.2 vs. 7.1 ± 1.6 vs. 4.1 ± 2.8 positive cells; cleaved caspase-3, 7.6 ± 2.1 vs. 19.6 ± 2.2 vs. 20.2 ± 4.1 positive cells; p-p38, 7.5 ± 2.4 vs. 16.3 ± 4.2 vs. 19.4 ± 4.4 positive cells, respectively; Figure 10, *p* < 0.05). These results further suggested that pitavastatin acted as an antiproliferative and proapoptotic agent in vivo.

## 3. Discussion

In the present study, our objective was to evaluate the anticancer potential of pitavastatin on cervical carcinoma by employing cervical cancer cell lines (Ca Ski, HeLa, and C-33 A) and a xenograft mouse model using C-33 A cells. First, we tested various concentrations and treatment durations of pitavastatin (5 and 10 μM; 24, 48 and 72 h, respectively) and found that each dose and treatment strongly reduced the viability and colony formation ability of cervical cancer cells, particularly HeLa and C-33 A cells (Figure 1A–E). Pitavastatin treatment also significantly inhibited cervical cancer xenograft animal tumor growth (Figure 9A–D). These results were in agreement with earlier studies showing that pitavastatin significantly prevented the proliferation of hepatocellular carcinoma [9,30], lung cancer [14], pancreatic cancer [31,32], ovarian cancer [33,34] and lymphoblastic leukemia cells [35,36]. This may be because pitavastatin is a lipophilic statin that can more easily enter cancer cells and has a longer half-life (approximately 11 h), which could more effectively inhibit mevalonate synthesis and even increase cancer cell death in comparison with hydrophilic statins [37,38]. Furthermore, we also observed that pitavastatin promoted cleaved PARP-1 activation; Abdullah et al. [37] and Jiang et al. [39] showed that pitavastatin blocks geranylgeranyl protein and mevalonate products to inhibit mevalonate pathway progression, leading to cleaved PARP accumulation and subsequent cancer cell apoptosis in ovarian cancer, breast cancer and glioblastoma.

In addition, our data demonstrated that pitavastatin induces apoptosis in cervical cancer cells via DAPI staining (cell fluorescence) or qualitative (flow cytometry, Figure 2) methods and that apoptosis regulation, as indicated by increases in the Bax/Bcl-2 ratio and cleaved caspase 3 protein expression, occurred at the same time as co-treatment with the caspase inhibitor z-DEVD-fmk (Figure 6, Figure 8 and Figure 10); these findings were consistent with the results of previous studies reported by Otahal et al. [40] and Dewider et al. [41], who showed that pitavastatin could induce cell death to overcome erlotinib resistance in non-small cell lung cancer cell (NSCLC) lines (A549, Calu6 and H1993) and breast cancer cell lines (MDA-MB-231 and MCF7), which also rely on caspase-mediated apoptotic pathways. Crescencio et al. [42] showed that the increase in necrotic cell death was greater than that in apoptotic cell death in Ca Ski and HeLa cells after atorvastatin, fluvastatin and simvastatin treatment, which was not consistent with our study results. These different results may be similar to those of Paškevičiūtė and Petrikaitė [43], who revealed that a higher concentration of statins induced cell death through necrosis, while a lower concentration induced apoptotic cell death.

Besides apoptosis, Tang et al. [11] recently revealed that pitavastatin treatment could induce autophagy-dependent ferroptosis cell death in a triple-negative breast cancer cell line (MDA-MB-231), and pitavastatin is a potential ferroptosis-inducing agent, Zaky et al. [5] and Tripathi et al. [16] indicating that apoptosis, autophagy and ferroptosis may represent the basis of anticancer progression potential adjuvant treatment by statins in various cancers. Furthermore, Almeida-Nunes et al. [7] and Gbelcová et al. [8] revealed that pitavastatin acts through competitive inhibition to prevent mevalonate production, leading to alteration of the cancer cellular lipid metabolism and blocking cholesterol biosynthesis, thereby disrupting the integrity of the cancer cell membrane structure and then inducing the disintegration of cancer cell spheroids. These processes will lead to increased sensitivity of cancer cells to combination treatment with chemotherapy, such as carboplatin, paclitaxel and cisplatin, and will help to prevent CD8^+^ T cell exhaustion to impede tumor growth and metastasis.

As reported in early studies, pitavastatin induced cell death by causing cell cycle arrest at the sub-G1 phase (MIA PaCa-2, Huh-7 and SMMC7721 cells) [13,31] or G0/G1 phase (ASPC-1, PANC-1, MDA-MB-231, MCF7, A375 and WM115 cells) [32,41,44]. In this study, pitavastatin also caused sub-G1 phase arrest in Ca Ski, HeLa and C-33 A cells and increased the percentage of Ca Ski and HeLa cells in the G0/G1 phase but not in C-33 A cells, which presented a greater percentage of cells in the G2/M phase (Figure 3). The differences observed in the cell cycle phase arrest may be caused by Ca Ski and HeLa cells showed shorter doubling times than C-33A cells (24–26 h for Ca Ski cells, 23–25 h for HeLa cells, 23–32 h for C-33A cells) [45], and HPV E6 and E7 oncoproteins able to override the G1/S-phase checkpoint (cyclin E/A-CDK2) and then to prevent damaged DNA to repair in HPV-positive transformed cells (Ca Ski and HeLa cells) [46,47,48]. In addition, Crescencio et al. [42] reported that in atorvastatin-, fluvastatin- and simvastatin-treated Ca Ski, HeLa and ViBo cells, cell cycle arrest occurred in the sub-G1 phase, and Sánchez et al. [49] also showed that these statins increased the proportion of MCF-7 cells in the G0/G1 and G2/M phases. Recently, Hacıseyitoğlu et al. revealed that low-dose pitavastatin (1 μM) inhibited the proliferation of HeLa cells by inducing cell cycle arrest in the sub-G1 and G2/M phases, whereas high-dose pitavastatin (10 and 100 μM) caused HeLa cells to be in the S phase [50]. These differences in cell cycle phase arrest resulting from pitavastatin treatment may be associated with differences in cancer cell type and the dose- or time-dependent bioavailability of pitavastatin.

Several studies have reported drug treatments that decrease mitochondrial ∆ψm and ROS production to induce apoptosis, resulting in cancer cell death in certain cancer cell lines, such as HepG2, CT26 and HCT116 [51,52,53]. We noted that treatment of Ca Ski and HeLa cells with pitavastatin significantly decreased mitochondrial ∆ψm, resulting in mitochondrial dysfunction, although C-33 A was not affected (Figure 5), which was consistent with our previous study indicating that pitavastatin induces apoptosis/necrosis in association with decreasing mitochondrial ∆ψm in MIA PaCa-2 cells [31]. Sánchez et al. [49] also showed that fluvastatin induced the loss of mitochondrial ∆ψm and subsequently the loss of mitochondrial membrane integrity in MCF-7 cells, but atorvastatin and simvastatin did not influence mitochondrial membrane integrity.

Regarding upstream signaling pathways, the PI3K/AKT signaling pathway plays an important role in regulating the development and progression of malignancies, and in common cancers, this pathway is overactive or aberrantly dysregulated, including in cervical cancer [27,28,54,55,56,57]. Hu et al. [14] revealed that pitavastatin suppresses Ras/Raf/MEK and PI3K/AKT/mTOR signaling to promote apoptosis and ameliorate angiogenesis in lung cancer cells. Similarly, Lee et al. [12] showed that pitavastatin promotes FOXO3a nuclear transcription mainly by inhibiting AKT and activating AMPK, resulting in apoptosis in oral cancer SCC15 cells. Recently, Elbaset et al. reported that pitavastatin reduces thioacetamide (TAA)-induced renal injury [58] and liver fibrosis [59] in rats by suppressing PI3K/AKT/mTOR signaling. These findings are consistent with the results from our study, which demonstrated that pitavastatin treatment inhibited PI3K/AKT in Ca Ski cells; however, p-AKT expression was not inhibited in HeLa and C-33 A cells (Figure 7), as previous studies have revealed that pitavastatin induces cardioprotective effects through the suppression of IKK/NF-κB and the upregulation of p-AKT-eNOS and AKT/GSK3β in failing rat hearts [60,61,62].

In addition, the inhibition of phosphorylated p38, ERK1/2, and JNK1/2 was observed in Ca Ski and HeLa cells following pitavastatin administration, in contrast to the lack of inhibition of p-ERK1/2 and p-JNK1/2 in C-33 A cells. These findings align with prior studies indicating the anti-inflammatory effects of pitavastatin on interleukin-1β-induced inflammation in SW982 human synovial sarcoma cells via the deactivation of p38, ERK, and JNK pathway phosphorylation [63]. Similarly, Kaushik et al. [64] reported that pitavastatin mitigated cisplatin-induced renal toxicity by deactivating the same pathways. However, Tsujimoto et al. and Kim et al. showed that pitavastatin enhances H_2_O_2_-induced apoptosis in VSMCs by increasing the phosphorylation of p-p38 and p-JNK [65], while pitavastatin-induced apoptosis in cutaneous squamous cell carcinoma cells (SCC12 and SCC13) occurs via activation of p-JNK and does not affect p-p38 [66]. These differential results for the pitavastatin-mediated signaling pathway may be dependent on the pitavastatin concentration, time stimulus and cell type. Ca Ski and HeLa cells were infected with HPV types 16 and 18, respectively [67], whereas C-33 A cells were HPV-negative cells that expressed the oncogenes p53 or pRb protein [68]. According to the above results, the presence or absence of HPV may be inconsistent because of its antiproliferation effects.

## 4. Materials and Methods

### 4.1. Chemicals

Both pitavastatin and Z-DEVD-FMK (caspase-3 inhibitor; #14414) were obtained from Cayman Chemical (Ann Arbor, Michigan, USA), and their purities were greater than 98% and 95%, respectively. They were dissolved in DMSO (D26650; Sigma-Aldrich, St. Louis, MO, USA) to prepare stock solutions (10 mM pitavastatin and 10 mM Z-DEVD-FMK) and were stored at −20 °C for further use.

### 4.2. Cell Culture

Three cervical cancer cell lines, Ca Ski, HeLa, and C-33 A, were obtained from the Bioresource Collection and Research Center (BCRC), Hsinchu, Taiwan. Ca Ski cells were cultured in RPMI-1640 medium (BioConcept, Amimed, Allschwil, Switzerland, Cat. No.: 1-41P05-K) supplemented with 10% fetal bovine serum (FBS, Cytiva, Cat. No.: SH30396.03). HeLa and C-33 A cells were maintained in Eagle’s MEM (Cytiva, Marlborough, MA, USA, Cat. No.: SH30024.02) supplemented with 10% FBS. All three cell lines were cultured according to the ATCC instructions.

### 4.3. Cell Cytotoxicity Assay

Cells were seeded in 96-well plates (1 × 10^4^ cells per well) and treated with pitavastatin (0, 5, or 10 µM) for 24, 48, or 72 h. The control group consisted of cells treated with 0.1% DMSO. Following treatment, CCK-8 solution (Dojindo Laboratories, Inc., Rockville, MD, USA, Cat. No.: CK04) was added to each sample, and the samples were kept in a cell culture incubator for 1 h. The absorbance at 450 nm was measured using a FLUOstar Galaxy microplate reader (BMG Labtech, Ortenberg, Germany).

### 4.4. Colony Formation

Cells (1 × 10^3^/well) were seeded in six-well plates and treated with 5 μM or 10 μM pitavastatin for 48 h. The incubation culture media were changed every 3 days. After 14 days, the cultures were fixed with 4% formaldehyde (in PBS) for 30 min, stained with crystal violet solution (0.1%) for 0.5 h, and imaged under an inverted microscope, after which the visible colonies were counted with ImageJ software (ver. 1.54c, http://rsb.info.nih.gov/ij, accessed on 15 March 2023, NIH, Bethesda, MD, USA).

### 4.5. DAPI Staining Assay

Cells (5 × 10^4^/well) were plated in 24-well plates. After adhesion for 24 h, the cells were treated with 5 μM or 10 μM pitavastatin for 48 h. Then, the cultures were fixed with 4% formaldehyde (in PBS) for 30 min, permeabilized with 0.1% Triton X-100 (in PBS) for 10 min and then stained with DAPI fluorescent dye (5 μM) for 30 min. The images were captured using a microscope equipped with an Olympus DP73 camera (Olympus Corporation, Shinjuku, Tokyo, Japan). For each cell sample, three fields at 400× magnification were imaged and assessed using the Image-Pro image analysis platform (Media Cybernetics ver. 4.5, Silver Spring, MD, USA).

### 4.6. Apoptosis and Cell Cycle Analysis

Cells were seeded in six-well plates (at 1 × 10^6^ cells per well) and cultured for 24 h. The cells were treated with 5 μM or 10 μM pitavastatin for 48 h. The cells were harvested and centrifuged at 3000 rpm for 10 min. For apoptosis analysis, a FITC Annexin V apoptosis detection kit (BD Biosciences, Bergen, NJ, USA, Cat. No.: 556547) was used. Briefly, the cells were suspended in 100 μL of 1× binding buffer. Then, 5 μL each of Annexin-V (20 μg/mL) and propidium iodide (50 μg/mL) were added to each sample. After that, the cells were incubated in the dark at 22 °C for 15 min. Next, binding buffer (900 μL/sample) was added, and the cells were then measured using a Cytomics FC500 flow cytometer to determine the percentages of both early and late apoptotic cells.

For the cell cycle analysis, the process of treating the cells with pitavastatin was the same as that described above. After cell harvesting, the cells were fixed with 70% ethanol chilled to −20 °C overnight. Next, the cells were centrifuged at 3000 rpm for 10 min at 4 °C. After they were washed, 500 μL of propidium iodide/RNase staining buffer (BD Bioscience, Cat. No.: 550825) were added, and the mixture was incubated for 15 min at ambient temperature in the dark. The samples were analyzed using a flow cytometer.

### 4.7. Wound Healing and Migration Assay

Cells were seeded in six-well plates (at 2 × 10^6^ cells per well). After 24 h, a wound was made in each well in the middle of the confluent monolayer of culture by using a 200-μL pipette tip. After being washed twice with PBS, the cultures were treated with 5 μM or 10 μM pitavastatin. Images were captured at 0, 24, and 48 h using an Olympus DP73 camera (Olympus Corporation) and subsequently analyzed using ImageJ software. For migration assay, cells (2 × 10^5^) were seeded in the upper transwell chamber including 8 μm pore size membrane filter (BD Biosciences) in serum-free medium, and the bottom chamber were contained serum standard medium. After being treated with 5 μM or 10 μM pitavastatin for 24 or 48 h, migration cells were fixed 100% methanol and stained with 0.1% crystal violet solution (Sigma-Aldrich, in PBS). To reduce the variability of the results, at least three observations were recorded for each well, and the area of the wound and migration numbers were used to evaluate the percentage relative to the untreated control group.

### 4.8. Mitochondria Membrane Potential Measurement

Cells were seeded in six-well plates (at 1 × 10^6^ cells per well) and grown in a 37 °C cell culture incubator overnight. The cells were then treated with 5 or 10 μM pitavastatin for 48 h. All samples were collected and stained with BD Pharmingen MitoScreen JC-1 dye (BD Bioscience, Cat. No.: 551302) for 15 min in a 37 °C cell culture incubator. The stained cells were harvested, resuspended in 0.5 mL of 1× assay buffer and detected by flow cytometry.

### 4.9. Immunoblotting Analysis

The cells were seeded in 10 cm dishes (total 2 × 10^6^ cells) and pretreated with or without z-DEVD-fmk (2.5 or 5 µM) for 2 h at 37 °C, after which the cells were exposed to various concentrations of pitavastatin (0, 5, or 10 μM) for 48 h. For protein analysis, cell lysates were prepared with RIPA buffer (Millipore, Billerica, MA, USA, Cat. No.: 20188), and the protein concentrations were determined using a BCA protein assay (Thermo Fisher, Waltham, Middlesex, USA, Cat. No.: 23225). A total of 30 µg of protein was separated on a 10–12% (*w*/*v*) SDS‒PAGE gel and transferred onto PVDF membranes (Bio-Rad, Irvine, CA, USA, Cat. No.: EA162-0177; pore size = 0.2 µm). Following the transfer, the membrane was blocked using BlockPRO protein buffer (Energenesis Biomedical, Taipei, Taiwan) for 1 h. Primary antibodies against PARP (Cat. No.: 9532), Bcl-2 (Cat. No.: 15071), Bax (Cat. No.: 5023), cleaved caspase-3 (Cat. No.: 9664), p27^Kip1^ (Cat. No.: 3686), phospho-AKT (*p*-AKT; Ser473, Cat. No.: 4060), Akt (Cat. No.: 4298), phospho-p38 (*p*-p38, Cat. No.: 9211), p38 (Cat. No.: 9212), phospho-ERK1/2 (*p*-ERK1/2, Cat. No.: 4370), ERK1/2 (Cat. No.: 4695), phospho-JNK (*p*-JNK, Cat. No.: 4668) and JNK (Cat. No.: 9258) (all from Cell Signaling Technology, Danvers, MA, USA); and PIK3CA (p110α) (Novus Cat. No.: NBP2-19804) and GAPDH (Thermo Fisher Cat. No.: MA5-15738) were used to probe the membranes at 4 °C overnight. Subsequently, an IgG polyclonal secondary antibody, either horseradish peroxidase (HRP)-conjugated goat anti-mouse (1:50,000) or anti-rabbit (1:100,000), was applied to the membrane for 1 h at ambient temperature. Finally, the signals on the membranes were visualized by using an enhanced chemiluminescence (Clarity MaxTM Western ECL) substrate (Clarity and Clarity Max, Bio-Rad, Cat. No.: 1705062), and the relative intensities of the signals were measured with Fusion FX7 system (version 16.08a; Labtech International, Inc., Vilber Lourmat, France).

### 4.10. Tumor Xenograft Mouse Model

Seven-week-old female BALB/cAnN Cg-*Foxn1^nu^*/CrlNarl (NUDE) mice weighing 20 ± 2 g were purchased from the National Laboratory Animal Center (Taipei, Taiwan). All animals were raised in a specific pathogen-free environment within a controlled environment featuring a 12:12-h light/dark cycle at 22 °C for 10 days. A total of 24 mice were divided into three groups (*n* = 8/group). C-33 A cells (1 × 10^7^) were embedded in Matrigel (Corning 354248, Tewksbury, MA, USA) at a ratio of 2:1 in PBS and then subcutaneously implanted into the right flank of the animals. Cancer cell implantations were performed under 2–3% isoflurane (Panion & BF Biotech, Taipei, Taiwan) inhalation, and maximal efforts were made to alleviate the suffering. When tumors reached a volume > 300 mm^3^ (day 9), the mice received daily intraperitoneal injections of pitavastatin (5 or 10 mg/kg), which was dissolved in a solution composed of DMSO, Cremophor (75% in ethanol) and PBS at a ratio of 1:4:5. After pitavastatin treatment for 14 days (day 23), the tumor-bearing nude mice were humanely euthanized via carbon dioxide inhalation, and the tumors were subsequently excised for tissue analysis. Throughout the study duration, the dimensions of the tumors were measured using a digital caliper, while the body weights of the subjects were recorded every other day. Tumor volumes were determined using the standard formula: tumor volume = 0.5 × [(length of tumor) × (width of tumor)^2^]. The animal experimental protocols received ethical approval from the Institutional Animal Care and Use Committee (IACUC, Changhua, Taiwan) at our institution (Approval ID: CCH-AE-111-007) and adhered to the 3Rs principle—replacement, reduction and refinement. 

### 4.11. Histology and Immunohistochemistry Analysis

Tumor tissue specimens were preserved using a 10% solution of neutral buffered formalin (Leica Biosystems, Richmond, IL, USA, Cat. No. 3800600) and dehydrated before being embedded in paraffin. The tumor specimens were subsequently cut into 5-µm sections and subjected to staining with hematoxylin and eosin (Leica Biosystems, Cat. No. 3801698) for immunohistochemical analysis. First, sections on slides were deparaffinized, followed by rehydration. After washing with PBS, the sections were boiled in ddH_2_O for 10 min and treated with 3% H_2_O_2_ for 10 min. The sections were incubated with 3% bovine serum albumin (Sigma‒Aldrich, St. Louis, MO, USA, Cat. No. A3294) in PBS for 1 h to prevent nonspecific binding. Subsequently, the slides were stained with ki67 (Cell Signaling Technology, Cat No. 12202; at a dilution of 1:400), cleaved caspase 3 (Cell Signaling Technology, Cat No. 9664; at a dilution of 1:100) and *p*-p38 (Cell Signaling Technology, Cat No. 9211; 1:200) antibodies at 4 °C overnight. Next, the sections were rinsed with PBS and stained with anti-mouse/rat/rabbit IgG HRP antibodies (GeneTex, Irvine, CA, USA, Cat. No. GTX83398) at ambient temperature for 30 min. Then, the sections were incubated with a DAB detection kit (GeneTex, Cat. No. GTX30939) at 37 °C for 20 min, after which they were counterstained with hematoxylin. Finally, images were captured (model BX6; Olympus Corporation), and four fields of each animal tissue sample were recorded at a magnification of 400×. The analysis was performed using the Image-Pro image analysis platform (Media Cybernetics ver. 4.5).

### 4.12. Statistical Analysis

The analysis of statistical data was conducted through the application of one-way analysis of variance (ANOVA) with subsequent comparison using Tukey’s post hoc test. This statistical approach was adopted for the purpose of conducting multiple group comparisons against a control group utilizing GraphPad Prism software (version 9.3.0; Dotmatics, San Diego, CA, USA). The results are presented as the mean ± standard deviation (SD) from no fewer than three separate experiments. A *p*-value less than 0.05 between groups was considered to indicate statistical significance.

## 5. Conclusions

In summary, the research presented here, with both in vitro and in vivo models, indicated that pitavastatin can significantly inhibit cervical cancer cell proliferation. This inhibition is facilitated by activation of the PI3K/AKT and ERK/JNK/p38 pathways, which induces intrinsic apoptosis within cervical cancer cells, causes cell cycle arrest at the sub-G1 phase, and restricts cell migration capabilities. Furthermore, in vivo experiments revealed that pitavastatin indirectly suppressed the development of xenograft tumors derived from cervical cancer cells but exhibited minimal to no cytotoxic effects. Based on these observations, pitavastatin has emerged as a potent candidate for cervical cancer therapy. Future research should further investigate this potential by incorporating clinical trials.

## Data Availability

The data collected and analyzed for this research are accessible through the corresponding author upon reasonable request.

## References

[1] Basu P., Mittal S., Vale D.B., Kharaji Y.C. (2018). Secondary prevention of cervical cancer. Best Pract. Res. Clin. Obstet. Gynaecol..

[2] Datta N.R., Stutz E., Liu M., Rogers S., Klingbiel D., Siebenhüner A., Singh S., Bodis S. (2017). Concurrent chemoradiotherapy vs. radiotherapy alone in locally advanced cervix cancer: A systematic review and meta-analysis. Gynecol. Oncol..

[3] Lin H., Wang D., Li H., Wu C., Zhang F., Lin Z., Yao T. (2022). Survival, treatment pattern, and treatment outcome in patients with cervical cancer metastatic to distant lymph nodes. Front. Oncol..

[4] Son J., Lin H.Y., Fu S., Biter A.B., Dumbrava E.E., Karp D.D., Naing A., Pant S., Piha-Paul S.A., Rodon J. (2023). Predictors of Oncologic Outcome in Patients Receiving Phase I Investigational Therapy for Recurrent or Metastatic Cervical Cancer. J. Immunother. Precis. Oncol..

[5] Zaky M.Y., Fan C., Zhang H., Sun X.-F. (2023). Unraveling the anticancer potential of statins: Mechanisms and clinical significance. Cancers.

[6] Hu Y.-B., Hu E.-D., Fu R.-Q. (2018). Statin use and cancer incidence in patients with type 2 diabetes mellitus: A network meta-analysis. Gastroenterol. Res. Pract..

[7] Almeida-Nunes D.L., Silvestre R., Dinis-Oliveira R.J., Ricardo S. (2023). Enhancing Immunotherapy in Ovarian Cancer: The Emerging Role of Metformin and Statins. Int. J. Mol. Sci..

[8] Gbelcová H., Rimpelová S., Jariabková A., Macášek P., Priščáková P., Ruml T., Šáchová J., Kubovčiak J., Kolář M., Vítek L. (2024). Highly variable biological effects of statins on cancer, non-cancer, and stem cells in vitro. Sci. Rep..

[9] Duggan S.T. (2012). Pitavastatin: A review of its use in the management of hypercholesterolaemia or mixed dyslipidaemia. Drugs.

[10] Yee L.L., Wright E.A. (2011). Pitavastatin calcium: Clinical review of a new antihyperlipidemic medication. Clin. Ther..

[11] Tang W.-J., Xu D., Liang M.-X., Wo G.-Q., Chen W.-Q., Tang J.-H., Zhang W. (2024). Pitavastatin induces autophagy-dependent ferroptosis in MDA-MB-231 cells via the mevalonate pathway. Heliyon.

[12] Lee N., Tilija Pun N., Jang W.J., Bae J.W., Jeong C.H. (2020). Pitavastatin induces apoptosis in oral squamous cell carcinoma through activation of FOXO3a. J. Cell. Mol. Med..

[13] You H.-Y., Zhang W.-J., Xie X.-M., Zheng Z.-H., Zhu H.-L., Jiang F.-Z. (2016). Pitavastatin suppressed liver cancer cells in vitro and in vivo. OncoTargets Ther..

[14] Hu T., Shen H., Huang H., Yang Z., Zhou Y., Zhao G. (2020). Cholesterol-lowering drug pitavastatin targets lung cancer and angiogenesis via suppressing prenylation-dependent Ras/Raf/MEK and PI3K/Akt/mTOR signaling. Anti-Cancer Drugs.

[15] Zhang Z.-Y., Zheng S.-H., Yang W.-G., Yang C., Yuan W.-T. (2017). Targeting colon cancer stem cells with novel blood cholesterol drug pitavastatin. Eur. Rev. Med. Pharmacol. Sci..

[16] Tripathi S., Gupta E., Galande S. (2024). Statins as anti-tumor agents: A paradigm for repurposed drugs. Cancer Rep..

[17] Elmore S. (2007). Apoptosis: A review of programmed cell death. Toxicol. Pathol..

[18] Pfeffer C.M., Singh A.T. (2018). Apoptosis: A target for anticancer therapy. Int. J. Mol. Sci..

[19] He Y., Sun M.M., Zhang G.G., Yang J., Chen K.S., Xu W.W., Li B. (2021). Targeting PI3K/Akt signal transduction for cancer therapy. Signal Transduct. Target. Ther..

[20] Engelman J.A. (2009). Targeting PI3K signalling in cancer: Opportunities, challenges and limitations. Nat. Rev. Cancer.

[21] Guo Y.J., Pan W.W., Liu S.B., Shen Z.F., Xu Y., Hu L.L. (2020). ERK/MAPK signalling pathway and tumorigenesis. Exp. Ther. Med..

[22] Braicu C., Buse M., Busuioc C., Drula R., Gulei D., Raduly L., Rusu A., Irimie A., Atanasov A.G., Slaby O. (2019). A comprehensive review on MAPK: A promising therapeutic target in cancer. Cancers.

[23] Nitulescu G.M., Van De Venter M., Nitulescu G., Ungurianu A., Juzenas P., Peng Q., Olaru O.T., Grădinaru D., Tsatsakis A., Tsoukalas D. (2018). The Akt pathway in oncology therapy and beyond. Int. J. Oncol..

[24] Sinkala M., Nkhoma P., Mulder N., Martin D.P. (2021). Integrated molecular characterisation of the MAPK pathways in human cancers reveals pharmacologically vulnerable mutations and gene dependencies. Commun. Biol..

[25] Choi Y., Park N.J.-Y., Le T.M., Lee E., Lee D., Nguyen H.D.T., Cho J., Park J.-Y., Han H.S., Chong G.O. (2022). Immune Pathway And Gene Database (IMPAGT) revealed the immune dysregulation dynamics and overactivation of the PI3K/Akt pathway in tumor buddings of cervical cancer. Curr. Issues Mol. Biol..

[26] Kanehisa M., Sato Y. (2020). KEGG Mapper for inferring cellular functions from protein sequences. Protein Sci..

[27] Chen Y.-H., Wu J.-X., Yang S.-F., Chen M.-L., Chen T.-H., Hsiao Y.-H. (2021). Metformin potentiates the anticancer effect of everolimus on cervical cancer in vitro and in vivo. Cancers.

[28] Chen Y.-H., Wu J.-X., Yang S.-F., Hsiao Y.-H. (2023). Synergistic combination of luteolin and asiatic acid on cervical cancer in vitro and in vivo. Cancers.

[29] Lee C.Y., Chen P.N., Kao S.H., Wu H.H., Hsiao Y.H., Huang T.Y., Wang P.H., Yang S.F. (2024). Deoxyshikonin triggers apoptosis in cervical cancer cells through p38 MAPK-mediated caspase activation. Environ. Toxicol..

[30] Kharouba M., El-Kamel A., Mehanna R., Thabet E., Heikal L. (2022). Pitavastatin-loaded bilosomes for oral treatment of hepatocellular carcinoma: A repurposing approach. Drug Deliv..

[31] Chen Y.-H., Chen Y.-C., Lin C.-C., Hsieh Y.-P., Hsu C.-S., Hsieh M.-C. (2020). Synergistic anticancer effects of gemcitabine with pitavastatin on pancreatic cancer cell line MIA PaCa-2 in vitro and in vivo. Cancer Manag. Res..

[32] Chen Y.H., Huang Y.C., Yang S.F., Yen H.H., Tsai H.D., Hsieh M.C., Hsiao Y.H. (2021). Pitavastatin and metformin synergistically activate apoptosis and autophagy in pancreatic cancer cells. Environ. Toxicol..

[33] Jawad M.J., Ibrahim S., Kumar M., Burgert C., Li W.-W., Richardson A. (2022). Identification of foods that affect the anti-cancer activity of pitavastatin in cells. Oncol. Lett..

[34] Jawad M.J., Richardson A. (2023). Ivermectin augments the anti-cancer activity of pitavastatin in ovarian cancer cells. Diseases.

[35] Piktel D., Nair R.R., Rellick S.L., Geldenhuys W.J., Martin K.H., Craig M.D., Gibson L.F. (2022). Pitavastatin is anti-leukemic in a bone marrow microenvironment model of b-lineage acute lymphoblastic Leukemia. Cancers.

[36] Piktel D., Moore J.C., Nesbit S., Sprowls S.A., Craig M.D., Rellick S.L., Nair R.R., Meadows E., Hollander J.M., Geldenhuys W.J. (2023). Chemotherapeutic activity of pitavastatin in vincristine resistant B-cell acute lymphoblastic leukemia. Cancers.

[37] Abdullah M.I., Abed M.N., Richardson A. (2017). Inhibition of the mevalonate pathway augments the activity of pitavastatin against ovarian cancer cells. Sci. Rep..

[38] Hussein B.H., Kasabri V., Al-Hiari Y., Arabiyat S., Ikhmais B., Alalawi S., Al-Qirim T. (2022). Selected statins as dual antiproliferative-antiinflammatory compounds. Asian Pac. J. Cancer Prev. APJCP.

[39] Jiang P., Mukthavaram R., Chao Y., Nomura N., Bharati I., Fogal V., Pastorino S., Teng D., Cong X., Pingle S. (2014). In vitro and in vivo anticancer effects of mevalonate pathway modulation on human cancer cells. Br. J. Cancer.

[40] Otahal A., Aydemir D., Tomasich E., Minichsdorfer C. (2020). Delineation of cell death mechanisms induced by synergistic effects of statins and erlotinib in non-small cell lung cancer cell (NSCLC) lines. Sci. Rep..

[41] Dewidar S.A., Hamdy O., Soliman M.M., El Gayar A.M., El-Mesery M. (2023). Enhanced therapeutic efficacy of doxorubicin/cyclophosphamide in combination with pitavastatin or simvastatin against breast cancer cells. Med. Oncol..

[42] Crescencio M.E., Rodríguez E., Páez A., Masso F.A., Montaño L.F., López-Marure R. (2009). Statins inhibit the proliferation and induce cell death of human papilloma virus positive and negative cervical cancer cells. Int. J. Biomed. Sci. IJBS.

[43] Paškevičiūtė M., Petrikaitė V. (2017). Differences of statin activity in 2D and 3D pancreatic cancer cell cultures. Drug Des. Dev. Ther..

[44] Al-Qatati A., Aliwaini S. (2017). Combined pitavastatin and dacarbazine treatment activates apoptosis and autophagy resulting in synergistic cytotoxicity in melanoma cells. Oncol. Lett..

[45] Mertens B., Nogueira T., Stranska R., Naesens L., Andrei G., Snoeck R. (2016). Cidofovir is active against human papillomavirus positive and negative head and neck and cervical tumor cells by causing DNA damage as one of its working mechanisms. Oncotarget.

[46] Scheffner M., Werness B.A., Huibregtse J.M., Levine A.J., Howley P.M. (1990). The E6 oncoprotein encoded by human papillomavirus types 16 and 18 promotes the degradation of p53. Cell.

[47] Moody C.A., Laimins L.A. (2010). Human papillomavirus oncoproteins: Pathways to transformation. Nat. Rev. Cancer.

[48] Rieckmann T., Tribius S., Grob T.J., Meyer F., Busch C.-J., Petersen C., Dikomey E., Kriegs M. (2013). HNSCC cell lines positive for HPV and p16 possess higher cellular radiosensitivity due to an impaired DSB repair capacity. Radiother. Oncol..

[49] Sánchez C.A., Rodríguez E., Varela E., Zapata E., Paez A., Massó F.A., Montaño L.F., Lopez-Marure R. (2008). Statin-induced inhibition of MCF-7 breast cancer cell proliferation is related to cell cycle arrest and apoptotic and necrotic cell death mediated by an enhanced oxidative stress. Cancer Investig..

[50] Hacıseyitoğlu A.Ö., Doğan T.Ç., Dilsiz S.A., Canpınar H., Eken A., Bucurgat Ü.Ü. (2024). Pitavastatin induces caspase-mediated apoptotic death through oxidative stress and DNA damage in combined with cisplatin in human cervical cancer cell line. J. Appl. Toxicol..

[51] Zhu Y.Y., Huang H.Y., Wu Y.L. (2015). Anticancer and apoptotic activities of oleanolic acid are mediated through cell cycle arrest and disruption of mitochondrial membrane potential in HepG2 human hepatocellular carcinoma cells. Mol. Med. Rep..

[52] Zhang J., Feng Z., Wang C., Zhou H., Liu W., Kanchana K., Dai X., Zou P., Gu J., Cai L. (2017). Curcumin derivative WZ35 efficiently suppresses colon cancer progression through inducing ROS production and ER stress-dependent apoptosis. Am. J. Cancer Res..

[53] Li J., Li T.-x., Ma Y., Zhang Y., Li D.-y., Xu H.-r. (2019). Bursopentin (BP5) induces G1 phase cell cycle arrest and endoplasmic reticulum stress/mitochondria-mediated caspase-dependent apoptosis in human colon cancer HCT116 cells. Cancer Cell Int..

[54] King D., Yeomanson D., Bryant H.E. (2015). PI3King the lock: Targeting the PI3K/Akt/mTOR pathway as a novel therapeutic strategy in neuroblastoma. J. Pediatr. Hematol./Oncol..

[55] Alzahrani A.S. (2019). PI3K/Akt/mTOR inhibitors in cancer: At the bench and bedside. Seminars in Cancer Biology.

[56] Chen Y.-H., Yang S.-F., Yang C.-K., Tsai H.-D., Chen T.-H., Chou M.-C., Hsiao Y.-H. (2021). Metformin induces apoptosis and inhibits migration by activating the AMPK/p53 axis and suppressing PI3K/AKT signaling in human cervical cancer cells. Mol. Med. Rep..

[57] Chen Y.-H., Wu J.-X., Yang S.-F., Yang C.-K., Chen T.-H., Hsiao Y.-H. (2022). Anticancer effects and molecular mechanisms of apigenin in cervical cancer cells. Cancers.

[58] Elbaset M.A., Mohamed B.M., Moustafa P.E., Esatbeyoglu T., Afifi S.M., Hessin A.F., Abdelrahman S.S., Fayed H.M. (2024). Renoprotective Effect of Pitavastatin against TAA-Induced Renal Injury: Involvement of the miR-93/PTEN/AKT/mTOR Pathway. Adv. Pharmacol. Pharm. Sci..

[59] Elbaset M.A., Mohamed B.M., Hessin A., Abd El-Rahman S.S., Esatbeyoglu T., Afifi S.M., Fayed H.M. (2024). Nrf2/HO-1, NF-κB and PI3K/Akt signalling pathways decipher the therapeutic mechanism of pitavastatin in early phase liver fibrosis in rats. J. Cell. Mol. Med..

[60] Kobayashi N., Takeshima H., Fukushima H., Koguchi W., Mamada Y., Hirata H., Machida Y., Shinoda M., Suzuki N., Yokotsuka F. (2009). Cardioprotective effects of pitavastatin on cardiac performance and remodeling in failing rat hearts. Am. J. Hypertens..

[61] Malik S., Sharma A.K., Bharti S., Nepal S., Bhatia J., Nag T.C., Narang R., Arya D.S. (2011). In Vivo Cardioprotection by Pitavastatin From Ischemic-reperfusion Injury Through Suppression of IKK/NF-κB and Upregulation of pAkt–e-NOS. J. Cardiovasc. Pharmacol..

[62] Nagaoka K., Matoba T., Mao Y., Nakano Y., Ikeda G., Egusa S., Tokutome M., Nagahama R., Nakano K., Sunagawa K. (2015). A new therapeutic modality for acute myocardial infarction: Nanoparticle-mediated delivery of pitavastatin induces cardioprotection from ischemia-reperfusion injury via activation of PI3K/Akt pathway and anti-inflammation in a rat model. PLoS ONE.

[63] Cheng B.-F., Gao Y.-X., Lian J.-J., Guo D.-D., Liu T.-T., Xie Y.-F., Wang L., Yang H.-J., Wang M., Feng Z.-W. (2017). Anti-inflammatory effects of pitavastatin in interleukin-1β-induced SW982 human synovial cells. Int. Immunopharmacol..

[64] Kaushik S., Tomar A., Puthanmadhom Narayanan S., Nag T.C., Arya D.S., Bhatia J. (2019). Pitavastatin attenuates cisplatin-induced renal injury by targeting MAPK and apoptotic pathways. J. Pharm. Pharmacol..

[65] Tsujimoto A., Takemura G., Mikami A., Aoyama T., Ohno T., Maruyama R., Nakagawa M., Minatoguchi S., Fujiwara H. (2006). A therapeutic dose of the lipophilic statin pitavastatin enhances oxidant-induced apoptosis in human vascular smooth muscle cells. J. Cardiovasc. Pharmacol..

[66] Kim K.-I., Kim S.-M., Lee Y.-Y., Lee Y., Kim C.-D., Yoon T.-J. (2023). Pitavastatin Induces Apoptosis of Cutaneous Squamous Cell Carcinoma Cells through Geranylgeranyl Pyrophosphate-Dependent c-Jun N-Terminal Kinase Activation. Ann. Dermatol..

[67] Pater M.M., Pater A. (1985). Human papillomavirus types 16 and 18 sequences in carcinoma cell lines of the cervix. Virology.

[68] Donat U., Rother J., Schäfer S., Hess M., Härtl B., Kober C., Langbein-Laugwitz J., Stritzker J., Chen N.G., Aguilar R.J. (2014). Characterization of metastasis formation and virotherapy in the human C33A cervical cancer model. PLoS ONE.

